# COL1A1 and SERPINE1 as Potential Therapeutic Targets in Diabetic Retinopathy: A Study Incorporating RNA Transcriptomics, Single‐Cell RNA Sequencing, and Proteomics

**DOI:** 10.1155/humu/3442342

**Published:** 2026-07-02

**Authors:** Xiaomei Nie, Gang Su, Ran Zhang, Han Wang, Mingbo Li, Qian Liang

**Affiliations:** ^1^ Department of Ophthalmology, The Second Affiliated Hospital of Zunyi Medical University, Zunyi City, Guizhou Province, China, zmc.edu.cn

**Keywords:** bioinformatics, COL1A1, diabetic retinopathy, molecular docking, SERPINE1

## Abstract

**Background:**

Diabetic retinopathy is caused by chronic hyperglycemia, which damages the retina′s blood vessels and neurons. This study is aimed at identifying potential therapeutic targets for DR.

**Methods:**

Transcriptomic and proteomic data were obtained from the Gene Expression Omnibus (GEO) and ProteomeXchange databases, respectively. Differentially expressed genes (DEGs) and differentially expressed proteins (DEPs) were intersected. An enrichment analysis of the overlapping genes was performed based on the DAVID database. A protein–protein interaction (PPI) network (STRING) was analyzed via Cytoscape/cytoHubba to identify key genes. Single‐cell RNA‐sequencing (scRNA‐seq) data were processed using Seurat. Gene set enrichment analysis (GSEA) (clusterProfiler) and molecular docking (EnrichR) were performed. High glucose‐induced retinal microvascular endothelial cells (RMECs) were used for functional assays.

**Results:**

The intersection of DEGs and DEPs yielded shared genes, enriched in the PI3K–Akt signaling pathway, AGE–RAGE signaling pathway in diabetic complications, complement and coagulation cascades, and ECM–receptor interaction; a PPI network incorporating these genes revealed two key DR‐associated highly expressed genes, COL1A1 and SERPINE1. GSEA showed that samples with high expression of these key genes were enriched in pathways such as reactome signaling by TGFB family members, inflammatory response, TGF‐*β* signaling, and reactome cell extracellular matrix interactions. Single‐cell and molecular docking analyses revealed high expression of the two key genes in fibroblasts and binding between SERPINE1 and paricalcitol, and HG induction increased their levels in RMECs, whereas knockdown of SERPINE1 repressed RMEC proliferation, migration, and invasion in vitro.

**Conclusion:**

This study identifies SERPINE1 and COL1A1 as possible DR therapeutic targets, providing new insights into relevant therapeutic development.

## 1. Introduction

Diabetic retinopathy (DR) is a microangiopathy caused by diabetes mellitus (DM) [1, 2]. The International Diabetes Federation reports that more than 10% of adults—around 500 million individuals worldwide—live with diabetes [3, 4]. Projections indicate that this number may increase to almost 800 million by the year 2045 [5]. Currently, the occurrence of DR is estimated to affect over 100 million people, with expectations to climb to 161 million by 2045 [6]. Over the last decade, great advances have been achieved in the diagnosis of DR, along with the evolution of therapeutic strategies for managing DR [7]. This has resulted in a decline in severe vision loss due to DM in some developing countries [8]. However, the steady increase in the number of people affected by DM has made it prudent to identify and timely prevent DR [8, 9]. At present, novel and reliable biomarkers are required for DR prediction and therapy [10].

Recent study in DR [11] applied multiomics analysis and single‐cell RNA sequencing (scRNA‐seq), which has revolutionized molecular profiling and enables high‐throughput analysis and comprehensive characterization of complicated biological systems [12, 13]. scRNA‐seq can characterize distinct cell populations in complex patient‐derived tissues, which has also strengthened our ability to quantify ligand–receptor expression across different cell types, thereby systematically elucidating intercellular communication networks [14–16]. In contrast, traditional bulk RNA sequencing (RNA‐seq), a technique often employed synergistically with scRNA‐seq in multiomics studies, offers a simpler, cost‐effective, and robust average expression profile while being unable to detect the difference between individual cells [17–20]. Proteomics refers to the entire proteome expressed in the genome of a specific cell or tissue, which is aimed at analyzing the composition, expression level, status of posttranslational modification, and interactions among the proteins based on a holistic point of view to understand the development of diseases [21]. By integrating the data of scRNA‐seq with multiomics analyses, novel biomarkers and potential therapeutic targets for DR can be identified, and the specific retinal cell type in the DR development can also be unraveled [12].

This study is aimed at systematically identifying potential biomarkers and therapeutic targets closely associated with the onset and progression of DR. To achieve this goal, we adopted an integrative multiomics strategy. We first systematically screened DR‐related molecular features at the transcriptomic, proteomic, and single‐cell levels, then gradually narrowed down key molecules through multilayered bioinformatics analyses, and finally performed preliminary functional validation of candidate targets using in vitro cell models. This research design moves stepwise from bulk expression profiles to single‐cell resolution and from computational prediction to experimental validation, striving to comprehensively unravel the molecular pathology of DR. The potential clinical value of this study lies in the prospect that the identified key genes may serve as novel biomarkers for early diagnosis of DR, thereby offering new directions for improving clinical management of the disease and carrying significant translational potential.

## 2. Analytical Methods

### 2.1. Data Source

#### 2.1.1. The RNA‐seq Dataset

The Gene Expression Omnibus (GEO) database (https://www.ncbi.nlm.nih.gov/geo/) [22, 23] was accessed to obtain DR RNA‐seq datasets, GSE102485 (retina samples from 22 DR patients and three healthy controls) and GSE160306 (retinal macular samples from 26 DR patients and 20 healthy controls).

#### 2.1.2. scRNA‐seq Dataset

The DR scRNA‐seq dataset (GSE165784, comprising fibrous membrane samples from five DR patients) was also downloaded from the GEO database [24].

#### 2.1.3. Proteomics Dataset

The DR proteomics dataset PXD025986 was downloaded from the ProteomeXchange database (https://www.proteomexchange.org/), and the vitreous samples were collected from 23 DR cases and 27 controls [25].

### 2.2. Differential Analysis and Functional Enriched Pathway Analysis

All samples were divided into case (DR) and control (healthy control) groups. Thereafter, using the R package “DESeq2,” DEGs between the two groups were selected with thresholds of |log_2_ *F* *C*| ≥ 1 and *p*.adj < 0.05 [26]. Differentially expressed proteins (DEPs), meanwhile, were identified using the “Kammers” algorithm of the R package “MSstats” at the thresholds of |log_2_ *F* *C*| ≥ 0.585 and *p*.adj < 0.05 [27]. The DEGs and DEPs were finally visualized in the heatmaps and were then intersected in a Venn diagram to identify the shared genes.

The DAVID database (https://davidbioinformatics.nih.gov/) was applied for the functional enriched pathway analysis on the shared genes of the aforementioned differential analysis, and the significantly enriched pathways were taken (*p* < 0.05) [28].

### 2.3. Protein–Protein Interaction (PPI) Network and Sorting of Key Genes

The shared genes were uploaded to the STRING database (https://cn.string-db.org/) to develop a PPI network at a minimum interaction score of 0.4. The resulting PPI network was then imported into Cytoscape (Version 3.8.0) and processed using the “cytoHubba” plugin to identify the Top 10 genes according to six algorithms, including degree, edge percolated component, maximal clique centrality, maximum neighborhood component, closeness, and radiality. The common genes across these algorithms were selected as the key genes, and their expression levels in DR cases and healthy controls were determined.

### 2.4. Gene Set Enrichment Analysis (GSEA)

Based on the median expression of the key genes, samples were divided into high‐ and low‐expression groups, and GSEA was conducted using the HALLMARK and REACTOME gene set collections.

### 2.5. Processing of scRNA‐seq Data

The “Seurat” R package was applied for the analysis [29]. In detail, the cells with the gene counts between 200 and 6000 and the mitochondrial gene count lower than 10% in the dataset GSE165784 were applied to identify cell clusters, followed by the standardization via the “SCTransform” function. The batch effects across the samples following the dimensionality reduction were removed using the “harmony” R package, and the uniform manifold approximation and projection (UMAP) dimensionality reduction was additionally conducted to recognize the main principal components (PCs) [30]. The Top 50 main PCs were finally applied for the plotting of the KNN plot based on the Euclidean distance, and the “FindCluster” function (at a resolution of 0.1) was adopted to cluster cell population. Using the “FindAllMarkers” function at logfc.threshold = 0.30, min.pct = 0.25, and only.pos = T, identified cell populations were annotated.

### 2.6. Molecular Cocking Analysis

Molecular docking between the key genes and drug candidates was performed using the “Enrichr” R package [31]. The UniProt database was accessed to download the crystal structures of receptor proteins corresponding to the identified key genes, which were prioritized to those derived from X‐ray, nuclear magnetic resonance (NMR), or electron microscopy (EM) and those with higher resolution (as indicated by the lower Å values). Meanwhile, the possible drug candidates for the key genes were summarized from the DSigDB database, whereas the 3D structures of the targeted drugs were downloaded as ligands from the PubChem database (https://pubchem.ncbi.nlm.nih.gov/) based on the ranking results of the *p* value and their identity as drugs, which were then processed for molecular docking. Docking results with binding energy < −5 kcal/mol and residue distance < 3.5 Å were retained.

### 2.7. Cell Culture and Modeling

The rat retinal microvascular endothelial cells (RMECs) were obtained from Cell Biologics (code: M1266, Chicago, Illinois, United States) and cultured in the specialized culture medium (CM‐R114, Procell System, Wuhan, China) as recommended by the producer at 37°C with 5% CO_2_. For the modeling, RMECs were grown in the medium with either control glucose (5 mM, G116306, Aladdin, Shanghai, China) or high glucose (25 mM) for 6 days with a partial medium change on Day 3. Cells from Passages 7 to 9 were applied for the analysis [32].

Prior to the modeling, the lentiviruses of small interfering RNA against SERPINE1 and the scramble controls were ordered from Genechem (Shanghai, China) and applied to infect the RMECs, which were then treated with puromycin (P432988, Aladdin, China) to screen the cells with stable knockdown of SERPINE1 for subsequent analyses [33].

### 2.8. Reverse‐Transcription Quantitative PCR

The total cellular RNA was extracted using an RNA extractor kit (T751379, Aladdin, China); a spectrophotometer was used to test the concentration. Thereafter, 1 *μ*g of the RNA sample was applied to synthesize the first‐strand cDNA with a relevant synthesis kit (H665509, Aladdin, China). Then, the PCR was initiated using the SYBR Green qPCR Mix assay kit (S751588, Aladdin, China) in a real‐time PCR thermocycler (ABI7500, ThermoFisher, Waltham, Massachusetts, United States). Using the method 2^−*ΔΔ*ct^, the mRNA was computed and normalized to glyceraldehyde‐3‐phosphate dehydrogenase (housekeeping control) [34]. The primers applied were available in Table S1.

### 2.9. Cell Proliferation Assay

Following transfection and culture in high glucose, at 24 h posttransfection, RMECs were harvested and seeded into 96‐well plates at a density of 2 × 10^3^ cells per well. After culturing for 24, 48, and 72 h, 10 *μ*L of cell counting kit‐8 (CCK‐8) reagent (C0037, Beyotime, Shanghai, China) was added to each well and incubated for an additional 4 h. The optical density value at 450 nm was read in the iMark microplate reader (Bio‐Rad, Hercules, California, United States).

### 2.10. Cell Migration Assay

The transfected and modeled RMECs were seeded in a six‐well plate and grown until fully confluent, and a 10‐*μ*L sterile pipette tip was used to make a scratch. Subsequently, the RMECs were cultured in serum‐free medium for 48 h and the monolayers were visualized under an optical microscope (Olympus, Tokyo, Japan) to calculate the wound closure degree.

### 2.11. Cell Invasion Assay

The transfected and modeled RMECs were plated in the upper Transwell chamber (3422, Corning Inc., Corning, New York, United States) precoated with the thawed matrix gel (B753082, Aladdin, China) and added with the serum‐free culture medium, whereas the lower chamber was filled with 700‐*μ*L culture media containing 10% serum as the chemoattractant. Subsequently, the invaded cells were fixed and dyed with 0.1% crystal violet (C196471, Aladdin, China) for 20 min at room temperature. An optical microscope (Olympus, Japan) was applied to observe the invaded cells.

### 2.12. Statistical Analysis

R software (Version 4.1.0) and GraphPad Prism (Version 10.6.1) were applied for the current statistical analysis. The Wilcoxon rank‐sum test was applied to compare the significant differences between two groups of continuous data for computational analysis. Data of experimental assays were compared by Student′s *t*‐test and two‐way analysis of variance (ANOVA). All data, unless specified, were statistically significant when the *p* value < 0.05.

## 3. Results

### 3.1. Identification of DEGs and DEPs in DR

**Figure 1:**
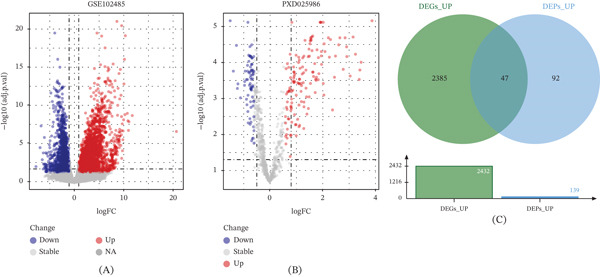
Differentially expressed genes (DEGs) and differentially expressed proteins in diabetic retinopathy. (A) The DEGs filtered at |log_2_ *F* *C*| ≥ 1 and *p*.adj < 0.05 from diabetic retinopathy in the dataset GSE102485. (B) The DEGs filtered at |log_2_ *F* *C*| ≥ 0.585 and *p*.adj < 0.05 from diabetic retinopathy in the dataset PXD025986. (C) The shared DEGs and differentially expressed proteins in diabetic retinopathy, as shown in the Venn diagram.

In the beginning, the DEGs and DEPs in DR were filtered and identified using the datasets GSE102485 and PXD025986 based on the specified thresholds, and the relevant volcano plots demonstrating the DEGs and DEPs were accordingly plotted in Figure 1A,B, respectively. Thereafter, the DEGs and DEPs with the upward expression trends were intersected in a Venn diagram, and 47 candidate genes were taken for follow‐up studies (Figure 1C, Table S2).

**Figure 2:**
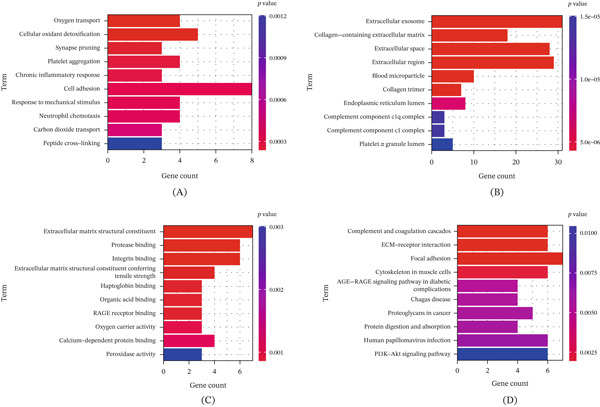
Functional enrichment analysis on the shared genes. (A–C) Results of gene ontology enrichment analysis on the shared genes based on the subcategories: biological processes, cellular components, and molecular functions. (D) Results of Kyoto Encyclopedia of Genes and Genomes enriched analysis on the shared genes.

Functional enriched pathway analysis was then performed on the shared DEGs via the DAVID database, and the shared DEGs were seen to be enriched in the pathways/processes like AGE–RAGE signaling pathway in diabetic complications, PI3K–Akt signaling pathway, complement and coagulation cascades, and ECM–receptor interaction (Figure 2A–D).

### 3.2. PPI Network and Sorting of Potential Key Genes

**Figure 3:**
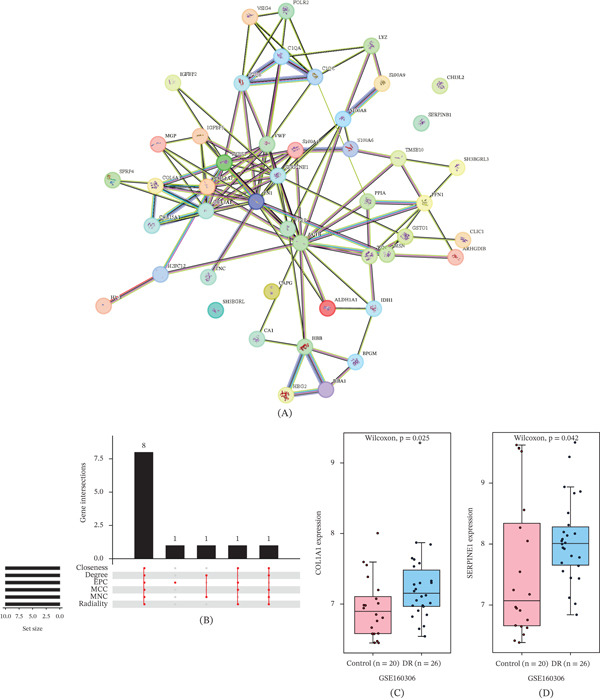
PPI network and sorting of the potential key genes for diabetic retinopathy. (A) The plotted PPI network based on the shared genes. (B) The upset plot showing the common genes of the six algorithms of the cytoHubba plugin of Cytoscape software. (C–D) Box plot of the expression levels of key genes COL1A1 and SERPINE1 in cases (diabetic retinopathy) and healthy controls.

The 47 shared DEGs were then uploaded to the STRING database to plot the PPI network, which was then visualized in Figure 3A. Thereafter, the PPI network was also uploaded to the Cytoscape software to screen the potential Top 10 key genes using the six algorithms of the “cytoHubba” plugin. The overlapping genes of each algorithm were subsequently intersected, and eight common genes were identified (Figure 3B). The expression levels of these eight genes were further validated in the independent validation set GSE160306. Among them, only two genes, COL1A1 and SERPINE1, exhibited a consistent upregulation trend in DR cases compared with controls, with statistical significance (Wilcoxon rank‐sum test, COL1A1: *p* = 0.025; SERPINE1: *p* = 0.042) (Figure 3C,D). There were no statistically significant differences in the remaining six genes between the DR group and the control group (*p* > 0.05). Therefore, we selected COL1A1 and SERPINE1 as key genes for further analysis.

### 3.3. GSEA on These Key Genes

**Figure 4:**
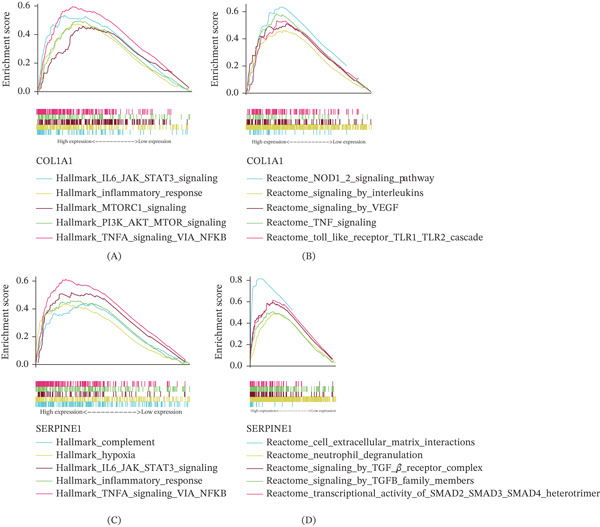
GSEA on the key genes COL1A1 and SERPINE1. (A–B) GSEA on the key gene COL1A1 based on the expression level. (C–D) GSEA on the key gene SERPINE1 based on the expression level.

Based on the median expression levels of COL1A1 and SERPINE1, samples were divided into the high‐/low‐expression group, and the GSEA was performed. The following pathways were enriched in samples with high expression levels of COL1A1 and SERPINE1: reactome signaling by TGFB family members, reactome cell extracellular matrix interactions, inflammatory response, reactome signaling by TGF‐*β* receptor complex, and TGF‐*β* signaling (Figure 4A–D).

### 3.4. Single‐Cell Landscape of DR

**Figure 5:**
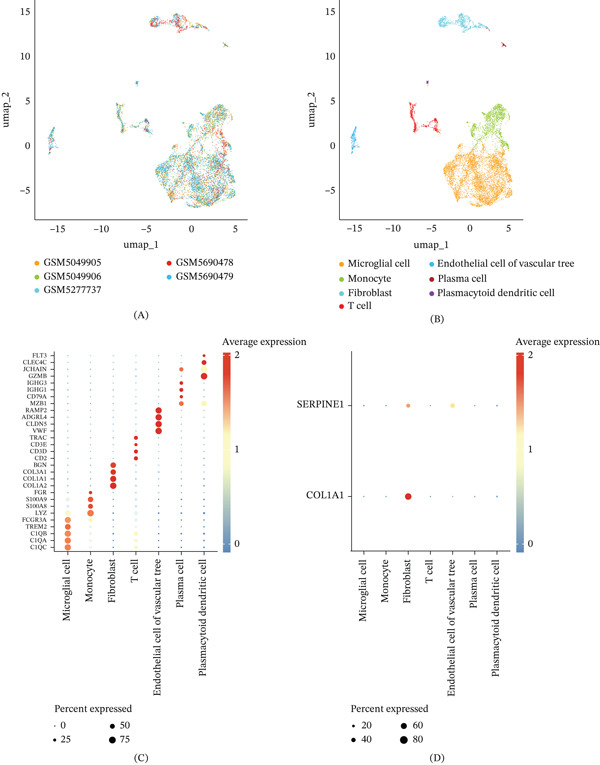
Single‐cell landscape in diabetic retinopathy of the dataset GSE165784. (A) UMAP plot demonstrating the single‐cell landscape in diabetic retinopathy from the dataset GSE165784 based on the samples. (B) UMAP plot of each annotated cell population (dots with colors) of the dataset GSE165784. (C) The expression levels of markers specific to each identified cell population in the dataset GSE165784. (D) COL1A1 and SERPINE1 expressions in the cell population identified.

The single‐cell landscape of the dataset GSE165784 in DR was plotted, and seven identified cell populations were annotated (Figure 5A–C): microglial cell (C1QC, C1QA, C1QB, TREM2, and FCGR3A), monocyte (LYZ, S100A8, S100A9, and FGR), fibroblast (COL1A2, COL1A1, COL3A1, and BGN), T cell (CD2, CD3D, CD3E, and TRAC), endothelial cell of vascular tree (VWF, CLDN5, ADGRL4, and RAMP2), plasma cell (MZB1, CD79A, IGHG1, and IGHG3), and plasmacytoid dendritic cell (GZMB, CLEC4C, and FLT3). The expression levels of the two key genes in the identified cell populations were determined, and their relatively high expression levels in fibroblasts were observed (Figure 5D).

### 3.5. Candidate Drug Predicted via Molecular Docking

**Figure 6:**
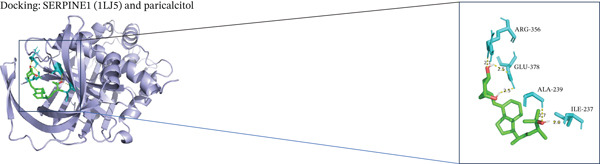
Molecular docking showing the stable binding between SERPINE1 and paricalcitol.

For the molecular docking, paricalcitol was selected as the candidate drug, and the protein crystal structures of the key genes COL1A1 (PDB ID: 1Q7D) and SERPINE1 (PDB ID: 1LJ5) were downloaded for the docking. The results revealed that the binding energy between SERPINE1 and paricalcitol was −7.56 kcal/mol (Figure 6).

### 3.6. Laboratory Validations on High Glucose‐Induced RMECs

For the laboratory validation, the RMECs were treated with normal glucose (control) or high glucose (model) and the expression levels of the key genes COL1A1 and SERPINE1 were quantified first, revealing a higher level in the modeled RMECs (Figure S1A). Following the confirmation of the knockdown efficiency via the lentiviruses (Figure S1B), the data of CCK‐8, scratch, and Transwell assays have shown that the silencing of SERPINE1 could repress the proliferation, migration, and invasion of high glucose‐induced RMECs in vitro (Figure S1C–E).

## 4. Discussion

In the current study, with the help of traditional RNA‐seq, scRNA‐seq, and proteomics, we highlight several important concepts that may account for the pathogenesis of DR. The relevant data have supported that the DEGs may be enriched in the processes like the PI3K–Akt signaling pathway, the complement and coagulation cascades, the AGE–RAGE signaling pathway in diabetic complications, and the ECM–receptor interaction. Meanwhile, two key DR‐associated genes COL1A1 and SERPINE1 were identified from the PPI network. The scRNA‐seq data further supported that these DR‐associated genes may express in fibroblasts and the molecular docking suggested that the binding between SERPINE1 and paricalcitol was stable. Collectively, these data provided a novel perspective for subsequent validation assays.

In the beginning, the data of traditional RNA‐seq and proteomics were summarized based on the datasets of GSE102485 and PXD025986, and the shared DEGs were applied for the enrichment analysis, and the pathways enriched were AGE–RAGE signaling pathway in diabetic complications, complement and coagulation cascades, ECM–receptor interaction, and PI3K–Akt signaling pathway. AGEs are advanced glycation end products generated by nonenzymatic modification of macromolecules by saccharides via the Maillard reaction. They can bind to receptors for AGE (RAGEs), altering innate and adaptive immune responses and thereby triggering inflammation and immunosuppression [35]. The role of such pathway in diabetes or diabetes‐mediated conditions has been extensively discussed, like diabetes‐mediated vascular calcification, diabetes osteoporosis, diabetic nephropathy, and more importantly, DR [36–38]. In the meantime, the PI3K/AKT pathway, which is activated due to the compensatory vascular hyperplasia in the late stage of DR, may result in the formation of fibrotic membranes during the development of DR [39]. The complement cascade, additionally, is imperative for ensuring the integrity and homeostasis of the retina, and the dysfunction of such cascade may cause certain retinal pathologies like DR [40]. ECM, or the extracellular matrix in the full form, serves as a platform for signaling networks and coordinates the cellular responses to environmental changes, which, however, may be excessively accumulated during DR to result in vascular remodeling, blood vessels stiffening, and impaired retinal function [41]. Similar enriched pathways were observed in another study that used the same dataset GSE102485 [42]. These data collectively substantiate the significance of these enriched pathways in DR.

Thereafter, the PPI network was plotted based on these shared DEGs and the key genes of the current study were identified as per the algorithms of the Cytoscape software, including COL1A1 and SERPINE1. Although existing studies have established COL1A1, a member of the collagen family, in oncology, several recent studies have also suggested its involvement in DR and its potential as a DR‐associated biomarker [43, 44]. SERPINE1 is another biomarker identified in the current study that may be associated with DR, and its oncogenic role has also been established in several publications [45, 46]. Similarly, the retinal neovascularization‐related genes, ferroptosis‐related biomarkers and autophagy‐related genes were also identified in the dataset GSE102485 [42, 47, 48]. In the current study, in addition to the finding that COL1A1 and SERPINE1 may serve as key genes in DR, the GSEA results revealed enrichment of reactome signaling by TGFB family members, reactome signaling by TGF‐*β* receptor complex, reactome cell extracellular matrix interactions, inflammatory response, and TGF‐*β* signaling in samples with high expression levels of COL1A1 and SERPINE1. Notably, the implications of the inflammatory response and TGF‐*β* signaling in DR have already been interpreted [49]. Such discoveries provided novel evidence on the potential molecular pathways involved in DR, thus warranting further validations. Furthermore, our molecular docking has suggested paricalcitol as the potential drug that could bind with SERPINE1. As a known synthetic vitamin D2 analog, paricalcitol can inhibit the secretion of parathyroid hormone via binding to the vitamin D receptor (VDR) [50]. In the research of diabetes or associated conditions, the combination therapy with enalapril and paricalcitol can attenuate streptozotocin‐induced testicular dysfunction in diabetes‐modeled rats via mitigating inflammation, apoptosis, and oxidative stress [51]. Another previous study has suggested that paricalcitol, as one of the VDR agonists, may attenuate the development of hyaloid vasculature, and pathological growth of ocular vasculature networks can underpin the visual impairment in DR [52].

However, it should be noted that this study has certain limitations. First, this study lacks in vivo functional validation in animal models; all conclusions are primarily based on in vitro cell experiments and bioinformatics analyses. Therefore, future research should establish animal models of DR (such as STZ‐induced diabetic rats or mice) to knock out or inhibit COL1A1 and SERPINE1 at the in vivo level, and observe their effects on retinal vascular lesions, inflammatory responses, and the fibrotic process, thereby more directly validating the pathogenic roles of these two genes. Second, the predicted interaction between paricalcitol and SERPINE1 is based solely on computational docking, without direct biophysical or functional validation. Subsequent studies should employ surface plasmon resonance (SPR) or isothermal titration calorimetry (ITC) to determine the binding affinity, as well as functional rescue assays (e.g., adding paricalcitol to high glucose‐treated RMECs to reverse the SERPINE1 high‐expression phenotype) to confirm the actual intervention efficacy. Third, the single‐cell data used in this study originated from fibrovascular membranes of patients with proliferative DR, representing end‐stage disease pathology; the cellular composition and molecular features may not fully reflect the status of early or nonproliferative DR; thus, caution is needed when extrapolating the conclusions to early‐stage DR. Future work should perform single‐cell sequencing on retinal or vitreous samples from DR patients at different stages to reveal the dynamic expression patterns and cellular origins of COL1A1 and SERPINE1 throughout disease progression. Moreover, the validation dataset in this study has a relatively small sample size (26 DR cases vs. 20 controls), which may affect statistical power and generalizability. Larger independent cohorts encompassing different ethnicities and DR stages are needed for further validation. Finally, the in vitro experiments only used RMECs, whereas the single‐cell data suggest that fibroblasts are the primary source of these two key genes. Future studies should therefore validate their functions in fibroblasts and establish coculture models of endothelial cells and fibroblasts to simulate the impact of cell–cell interactions on the expression of these key genes in DR. In summary, although this multiomics integrative study provides preliminary evidence supporting COL1A1 and SERPINE1 as potential targets in DR, the above limitations need to be systematically addressed through well‐designed in vivo and in vitro experiments as well as larger clinical cohort studies, thereby facilitating the translation of these findings toward clinical applications.

## 5. Conclusion

In summary, although our current study relies on bioinformatics data, it has elucidated potential pathogenic mechanisms underlying DR. The techniques of traditional RNA‐seq, the scRNA‐seq, and proteomics as well as the molecular docking have identified the relevant DEGs, key cell populations, and key genes associated with DR. The current study, we hope, may deepen our understanding on DR‐associated mechanisms and pave the way for discovering novel therapeutic approaches for DR in the future.

**Nomenclature**
CCK‐8: cell counting kit‐8
DEGs: differentially expressed genes
DEPs: differentially expressed proteins
DM: diabetes mellitus
DR: diabetic retinopathy
FC: fold change
GEO: Gene Expression Omnibus
GSEA: gene set enrichment analysis
NMR: nuclear magnetic resonance
PCs: principal components
PPI: protein–protein interaction
RMECs: retinal microvascular endothelial cells
RNA‐seq: RNA sequencing
scRNA‐seq: single‐cell RNA sequencing
UMAP: uniform manifold approximation and projection
VDR: vitamin D receptor

## Author Contributions

Xiaomei Nie oversaw the overall study design, project coordination, and manuscript composition. She also conducted the final critical review of the manuscript and served as the primary point of contact for editorial correspondence. Gang Su, Ran Zhang, Han Wang, Mingbo Li, and Qian Liang contributed to experimental execution, data curation and analysis, and the revision of the manuscript for intellectual content.

## Funding

This work was supported by Guizhou Science and Technology Cooperation Fundamentals (No. ZK [2023] General 529), and Guizhou Provincial Health Commission (Youth Project Category B) [Grant No. gzwkj2026‐367]

## Ethics Statement

Ethical approval was not required for this study because it is not involved in any human or animal experiments.

## Consent

The authors have nothing to report.

## Conflicts of Interest

The authors declare no conflicts of interest.

## Supporting Information

Additional supporting information can be found online in the Supporting Information section.

## Supporting information


**Supporting Information 1** Figure S1: Laboratory assays exploring the impact of SERPINE1 knockdown on HG‐induced RMECs. (A) The quantification of the mRNA levels of key genes COL1A1 and SERPINE1 in retinal microvascular endothelial cells cultured in normal or high glucose. (B) The validation on the SERPINE1‐specific lentiviruses on retinal microvascular endothelial cells. (C) The cell counting kit‐8 assay demonstrating the proliferation of the HG‐induced RMECs. (D) The scratch assay showing the migration of the HG‐induced RMECs at 0 and 48 h. (E) The Transwell assay demonstrating the invasion of the HG‐induced RMECs at 48 h.  ^∗∗^ or ^##^ *p* < 0.01, ^####^ *p* < 0.0001, and ^ns^ *p* > 0.05.

**Supporting Information 2** Table S1: Primers of the reverse‐transcription quantitative PCR assay.

**Supporting Information 3** Table S2: The 47 shared upregulated genes between DEGs and DEPs in diabetic retinopathy.

## Data Availability

The datasets generated and/or analyzed during the current study are available in the [GSE102485] repository, [https://www.ncbi.nlm.nih.gov/geo/query/acc.cgi?acc=GSE102485], [GSE160306] repository, [https://www.ncbi.nlm.nih.gov/geo/query/acc.cgi?acc=GSE160306], and [GSE165784] repository, [https://www.ncbi.nlm.nih.gov/geo/query/acc.cgi?acc=GSE165784].
