# Supplementary figures and images for: COL1A1 and SERPINE1 as Potential Therapeutic Targets in Diabetic Retinopathy: A Study Incorporating RNA Transcriptomics, Single‐Cell RNA Sequencing, and Proteomics
Source: Hum Mutat. 2026 Jul 2;2026:3442342. doi: 10.1155/humu/3442342 (PMC13329112; doi:10.1155/humu/3442342)

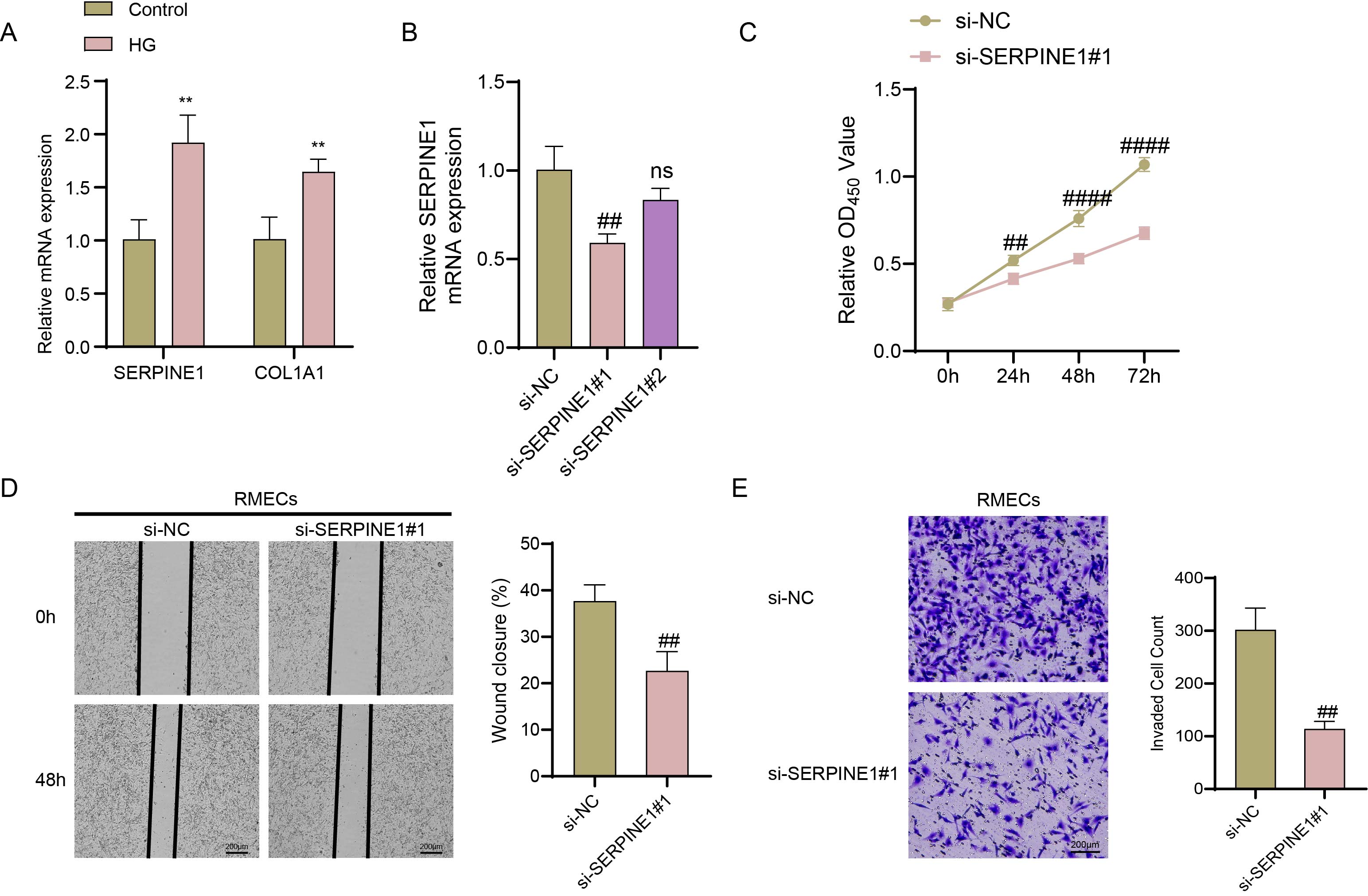

Supplement: Supplementary file 1 — Supporting Information 1 Figure S1: Laboratory assays exploring the impact of SERPINE1 knockdown on HG‐induced RMECs. (A) The quantification of the mRNA levels of key genes COL1A1 and SERPINE1 in retinal microvascular endothelial cells cultured in normal or high glucose. (B) The validation on the SERPINE1‐specific lentiviruses on retinal microvascular endothelial cells. (C) The cell counting kit‐8 assay demonstrating the proliferation of the HG‐induced RMECs. (D) The scratch assay showing the migration of the HG‐induced RMECs at 0 and 48 h. (E) The Transwell assay demonstrating the invasion of the HG‐induced RMECs at 48 h. ∗∗ or ## p < 0.01, #### p < 0.0001, and ns p > 0.05. [file HUMU-2026-3442342-s001.jpg]
