# Supplementary material for: COL1A1 and SERPINE1 as Potential Therapeutic Targets in Diabetic Retinopathy: A Study Incorporating RNA Transcriptomics, Single‐Cell RNA Sequencing, and Proteomics
Source: Hum Mutat. 2026 Jul 2;2026:3442342. doi: 10.1155/humu/3442342 (PMC13329112; doi:10.1155/humu/3442342)
Supplement: Supplementary file 2 — Supporting Information 2 Table S1: Primers of the reverse‐transcription quantitative PCR assay. [file HUMU-2026-3442342-s002.docx]

**Supplementary Table 1. Primers of the reverse-transcription quantitative PCR assay**

| Gene | Primers (5’-→-3’) | |
| --- | --- | --- |
|  | Forward | Reverse |
| Serpine1 | ACTTCTCAGAGATGGAAAGA | TCAGTCTCCAGAGAGAACTT |
| Col1a1 | CAGAAGTCATAGGAGTCGAG | TCTCATCATAGCCATAGGAC |
| Gapdh | TGAGTATGTCGTGGAGTCTA | CACAAAGTTGTCATTGAGAG |
